# An examination of validity and reliability of the Parental Stress Scale in a population based sample of Norwegian parents

**DOI:** 10.1371/journal.pone.0242735

**Published:** 2020-12-02

**Authors:** Ane Nærde, Silje Sommer Hukkelberg

**Affiliations:** The Norwegian Center for Child Behavioral Development, Oslo, Norway; Monash University, AUSTRALIA

## Abstract

The parental stress scale (PSS) is a widely used instrument that assesses stress related to child rearing. Even though several studies have investigated the construct validity and reliability of the PSS, no consensus has been reached regarding which and how many of the original eighteen items that should be included, or a robust factor structure with satisfactory reliability. The present study tested the psychometric properties of the Norwegian version of the PSS and used the advantages of complementary exploratory and confirmatory factor analyses to investigate the underlying factor structure of the PSS items. Data stem from a community sample of 1096 parents from five counties in Norway with a one-year-old child. The sample was randomly split (*N* = 553/543), and exploratory and confirmatory analyses were performed on each of the samples. Using predefined criteria for the selection of robust items, results revealed a two-dimensional structure (Parental stressors and Lack of rewards) across 13 PSS items, displaying satisfactory reliability. Network analyses revealed differential associations within item constellations and with covariates. Implications of the findings and study limitations are discussed.

## Introduction

Parenthood is at once rewarding and demanding. Indeed, the debate about the degree to which parents are more or less happy and healthy than nonparents, have engaged psychologists, sociologists, and economists alike for decades. The discussion was recently revitalized following a series of papers addressing parental happiness [1–3], including a comprehensive review of the mixed and conflicting literature [4]. Nelson and colleagues [4] concluded that the relationship between parenthood and happiness is complex, and that we need to ask *when*, rather than *if*, parenthood is associated with more happiness. Parents seem to report unhappiness when they experience negative emotions, financial problems, sleep difficulties, and troubled marriages, and happiness when having children bring greater meaning in life, satisfaction of basic needs, positive emotions, and enhanced social roles [4]. Importantly, we need to continue examining the circumstances under which parenthood is associated with more or less happiness.

### The concept of parental stress

The practical and emotional demands of child rearing and caregiving can undoubtedly be challenging and stressful; be it handling a crying 6-months-old, an unruly two-year-old, or an oppositional fourteen-year-old. *Parenting stress* is conceptualized as a negative psychological response to the numerous obligations associated with raising children and its presence is the rule rather than the exception [5–8]. Central to most definitions of parenting stress is the perceived balance between the practical and emotional requirements of parenting and the resources available for meeting them [7]. When the demands exceed the resources, parent’s typically experience high levels of stress. Deater-Deckard [8] defined parenting stress as the aversive psychological reaction to the demands of being a parent, which involves a complex process linking (a) the task demands of parenting, (b) the parent’s psychological well-being and behavior, (c) the qualities of the parent-child relationship, and (d) the child’s psychosocial adjustment. Feeling overwhelmed, incompetent in the parenting role, or consistently unhappy with one’s life can all be symptoms of parenting stress [9]. Whereas parental stress represents a normal consequence of parenting, levels of manageable stress may vary [8]. When parents experience enduring exposure to chronic parental stress, they are at the extreme end and at risk for *parental burnout* [10, 11], which is a prolonged response characterized by parental ineffectiveness, overwhelming exhaustion in the parental role, and emotional distancing from one’s children.

### The impact of parental stress

Substantial literature show that parenting stress is significantly associated with the well-being and adjustment of both parents and children (i.e., adult functioning, the quality of parent-child relationships, and child behavior and development). The research is broad, and addresses various types of parental stress across different samples, including a) minor stresses that are normal and frequent among parents of typically developing children (i.e., daily parenting hassles; [6, 12], b) general parenting stress in both non-clinical and clinical samples [7, 13], and c) parenting stress specific to having children with behavior problems, chronic illness, or developmental disabilities [14, 15]. Parental stress is linked with psychopathology (in particular maternal depression), sense of self-efficacy in the parenting role, parenting behavior (i.e., sensitivity, involvement, and intrusiveness), as well as co-parenting processes, and the marital relationship [16]. Moreover, parental stress has repeatedly been identified as a risk factor for child internalizing and externalizing behavior problems [6, 12, 17, 18]. In addition, recent innovative research have addressed the possibility that parental stress impact child development via epigenetic processes [19], although this research is still in its early days.

The mechanisms by which parental stress is linked with maladaptive behavioral and psychological functioning in children and parents are unclear. Already two decades ago, Deater-Dekard [8] emphasized the bi-directionality of parent-child interactions with regard to parental stress. Nevertheless, studies have typically addressed predictors or outcomes of parental stress rather than the reciprocal parent-child relationships [but see 17, 20–22]. The need to consider parental stress as bi-directional and multifaceted was recently highlighted by Deater-Deckard and Panneton [23] presenting current perspectives on parental stress and child development. In the same vein, Crnic and Ross [16] argued that the failure to treat parental stress as a systemic construct that reflects reciprocal and developmental processes within the family rather than an attribute or response of a single parent, represents a major shortcoming in the literature.

### The Parental Stress Scale (PSS)

The measurement of parental stress and the ability of assessment instruments to tap this complex concept is crucial for the quality of research [8, 16]. Among the relatively few instruments that assess differences in general parenting stress, the Parental Stress Scale (PSS) was developed to capture individual levels of stress associated with raising children [24]. This 18-item self-report measure (see Table 1 for item overview) holds advantages in that it is brief and easy to administer, and freely available [24, 25]. The PSS was originally developed as an alternative to the Parenting Stress Index (PSI) [5], which is lengthy and highly invasive, especially for non-clinical populations. The PSS focuses on parent’s perceptions of their parental role rather than the *sources* of stress. Importantly, Berry and Jones [24] wanted to address both the pleasures and strains associated with parenthood. Items were selected based on the so-called “conservation of resource model” [26], and loss in resources are reflected in items addressing for instance time, energy, or loss of control, whereas gains are reflected in items addressing for example happiness, closeness, and affection [24]. The items are rated on a Likert scale, and the final score of parental stress is obtained by adding all items together (positive items are reversed). Altogether, the PSS is designed to take a broad and unidimensional approach, and the index sum score is supposed to reveal if the costs outweigh the rewards.

**Table 1:** Factor solutions for the Parental Stress Scale (PSS) across studies.

Item		Berry & Jones 1995^1^ (PSS16)	Oronoz et al. 2007^2^ (PSS12)	Algarvio et al. 2018^3^ (PSS15)	Pontoppidan et al. 2018^4^ (PSS16)	Cheung 2000^5^ (PSS17)
	
PS01	I am happy in my role as a parent*	PREW	BREW	Not used	LPSAT	PSAT
PS02	There is little or nothing I wouldn’t do for my child(ren) if it was necessary*	NS	NS	Not used	Not used	Not used
PS03	Caring for my child(ren) sometimes takes more time and energy than I have to give	PSTRESS	PSTRESS	F/A	PSTRESS	PSTRAIN
PS04	I sometimes worry whether I am doing enough for my child(ren)	NS	NS	F/A	PSTRESS	PSTRAIN
PS05	I feel close to my child(ren)*	PREW	BREW	PSAT	LPSAT	PSAT
PS06	I enjoy spending time with my child(ren)*	PREW	BREW	PSAT	LPSAT	PSAT
PS07	My child(ren) is (are) an important source of affection for me*	PREW	Not used	PSAT	LPSAT	PSAT
PS08	Having children gives me a more certain and optimistic view for the future*	PREW	NS	PSAT	LPSAT	PSAT
PS09	The major source of stress in my life is my child(ren)	PSTRESS	PSTRESS	PSTRESS	PSTRESS	PSTRAIN
PS10	Having children leaves little time and flexibility in my life	PSTRESS	PSTRESS	PSTRESS	PSTRESS	PSTRAIN
PS11	Having children has been a financial burden	PSTRESS	PSTRESS	PSTRESS	Not used	PSTRAIN
PS12	It is difficult to balance different responsibilities because of my child(ren)	PSTRESS	PSTRESS	PSTRESS	PSTRESS	PSTRAIN
PS13	The behavior of my child(ren) is often embarrassing or stressful to me	PSAT	PSTRESS	PSTRESS	PSTRESS	PSTRAIN
PS14	If I had it to do over again, I might decide not to have children	LCONT	NS	LCONT	PSTRESS	PSTRAIN
PS15	I feel overwhelmed by the responsibility of being a parent	LCONT	PSTRESS	LCONT	PSTRESS	PSTRAIN
PS16	Having children has meant having too few choices and too little control over my life	PSTRESS / LCONT	Not used	LCONT	PSTRESS	PSTRAIN
PS17	I am satisfied as a parent*	PSAT	BREW	Not used	LPSAT	PSAT
PS18	I find my child(ren) enjoyable*	PREW / PSAT	BREW	Not used	LPSAT	PSAT

Note.

*Items that should be reversed; PREW: Parental rewards; PSTRESS: Parental stressors/stress; PSAT: Parental satisfaction; PSTRAIN: Parental strain; NS: Non-significant loading; LCONT: Lack of control; BREW: Baby’s Rewards; F/A: Fears/anxiety; LPSAT: Lack of parental satisfaction.

^1, 5^Principal axis factor analysis (Varimax rotation).

^2^Exploratory factor analysis (EFA) GLM (Oblimin rotation).

^3^Confirmatory factor analysis (CFA).

^4^Rasch modeling.

The PSS is a much-used instrument in both research and clinical practice. According to Louie, Cromer, and Berry [25], who reviewed two decades of research involving the PSS across 25 studies, the scale is currently translated into 26 languages. Its wide application within research encompasses different populations, including first-time parents [27, 28], parents of children with chronic somatic health conditions [29] and autism spectrum disorder [30, 31], population based samples [32], and a mix of clinical and non-clinical samples [33]. Whereas the number of valid items and factors varies between studies (see Table 1), Louie et al. [25] maintain that the PSS items represent a unidimensional scale. The range of modifications made across the various studies relate to for instance cultural differences in translating, variations in response format, sample characteristics, and statistical justifications.

The initial validation study by Berry and Jones [24] evaluated psychometric properties of the 18-items PSS in several steps using various groups of respondents. Following a principal axis factor analysis, four correlated factors across altogether 16 PSS items were identified; Parental rewards, Parental stressors, Loss of control, and Parental satisfaction [24] (see Table 1). Thus, already in the original study, two items were excluded due to lack of significant loadings. In addition, two significant cross-loadings were identified. This particular factor structure was tested and replicated by Zelman & Ferro [29] in a small sample of parents of children with chronic somatic illnesses aged 6–16 years (*N* = 50, *M*_age_ = 11.4). Still, other studies testing the structural validity of the PSS have concluded differently (see Table 1 for overview). For instance, Oronoz et al. [28] examined the psychometric properties of the Spanish version of the PSS among first-time parents with a baby between 3 and 8 months (*N* = 211, *M*_age_ = 5.37) using exploratory factor analyses (EFA) and obtained a two-factor solution (i.e., Stressors and Baby’s rewards) across 15 items. Moreover, Algarvio and colleagues [32] investigated a Portuguese version of the PSS in a large sample of parents with children attending public preschools and primary schools (*N* = 3842, *M*_age_ = 7.06). While results from confirmatory factor analyses (CFA) indicated a four-factor solution (Fears/anxiety, Parental satisfaction, Parental stressors, and Lack of control) across 14 items, the items labeling and the items included in each factor diverged from those of Berry and Jones [24].

Addressing the psychometric properties of a Chinese version of the PSS, Cheung [34] utilized a sample of adjusted and maladjusted families (i.e., with and without parent-child relationship problems) with at least one child younger than 12 years (*N* = 257, information on child age not provided). The results from a principal component analysis indicated a two-factor solution (Parental satisfaction and Parental strain) across 16 items, using a six-point response format rather than the original five. This Chinese version was also utilized by Leung and Tsang [33] to test the unidimensionality of the PSS using Rasch modeling in a non-clinical sample of parents with primary school children (*N* = 162, *M*_age_ = 9.24), and a small sample of parents having children with ADHD (*N* = 38, *M*_age_ = 8.54). They identified a 16-item version with the original five categories as unidimensional [33]. Moreover, Pontoppidan et al. [27] recently tested a Danish version of the PSS using Rasch modeling in a large sample of first-time mothers of 0 to 12-month-olds (*N* = 1110, *M*_age_ = 2.70). The results contrasted those of Leung and Tsang [33] in showing that the PSS consists of two separate subscales (Parental stress and Lack of parental satisfaction) across 17 items. Altogether, most studies advocate a multidimensional structure of the PSS (most often two) encompassing from 12 to 17 items (see Table 1). Given the widespread use of the PSS within research and clinical work, it is vital to continue exploring the scale’s validity, reliability and factor structure across different samples and contexts by the use of rigorous tests.

### Aims of the study

Given that parental demands vary across the course of childhood and across different groups of parents, there is a need to investigate the use of the PSS in different subgroups of parents, cultures, and settings. The PSS is fairly short, non-invasive, and freely available, making it suitable for use with parents of typically developing children. Very few studies have examined the use of PSS among parents of young infants. The present study aimed to investigate the reliability and validity of the PSS in a large community sample of Norwegian parents with a one-year-old child. To the best of our knowledge, this is the first study to examine the psychometric properties of the Norwegian version of the PSS, and the second in a Scandinavian country. We evaluate the PSS using two methodological approaches, that is, a latent variable approach and a network approach. Thus, we add to the field by providing empirical evidence about the dimensionality and psychometric properties of the PSS based on up-to-date psychometric analyses.

Since previous research has revealed different factor structures across various numbers and constellations of PSS items, we sought to examine the latent PSS construct from its origin, rather than replicate the previously described models. To uncover a robust factor structure, we first defined criteria for a parsimonious factor model (see method section). EFA and CFA were applied in two randomly split samples to uncover and confirm the PSS factor structure, respectively. To determine convergent validity, we examined the associations between the PSS and parent reported psychological distress (symptoms of depression and anxiety). A priori, we hypothesized that higher levels of parental stress are associated with more anxiety and depression.

Next, we investigated parental stress using a network approach. Different from the latent “common cause” methodology, which e.g., assumes that stress indicators are reflective and interchangeable items caused by a common underlying cause, the network approach considers that the different stress items influence and associate with each another. This web of stress *constitute* parental stress. As such, the network approach provides a different understanding of the parental stress construct. In the network, stress symptoms are presented as *nodes*, and the connections between them as *edges* [35]. The edges provide information about the quality of the connections through their thickness and color. The more two items associate, the thicker is the edge between them. A blue edge reflects a positive association, whereas a red edge reflects a negative. In addition, nodes with many connections, which more likely spread activation throughout the network, are situated more central compared to those with fewer connections [35, 36]. The present study provides a first attempt to an alternative understanding of parental stress, as consisting of central and peripheral stress items that connect and interact.

## Materials and methods

### Participants

The participants came from the Behavior Outlook Norwegian developmental Study (BONDS), wherein 1157 children (559 girls) and their families have been followed from the children were 6 months old throughout early school age [37]. The children were born between 2006 and 2008, and families were recruited at the regular 5-month check-up at local child health clinics, which are public, free, and almost universally attended. Families of 1,931 eligible children who lived in one of five counties situated in two Norwegian municipalities were informed about the study. Apart from the child’s age, the inclusion criterion was that minimum one parent could participate without the need of a translator. Altogether 1,465 (76%) families agreed to be contacted, and 1,159 (60%) wanted to participate. Families of two children later decided to opt out of the study and have all data deleted, and the final sample size is 1157.

The study sample is comparable to the informed families on key background variables with the exception of higher education among participating mothers [37]. Compared to the Norwegian population of newborns in the same period, the study sample has more firstborns (47% vs. 43%), fewer mothers born outside Norway (i.e., both from Europe [7% vs. 10%] and outside Europe [6% vs. 12%]), fewer single mothers (5% vs. 11%), and higher educated mothers (i.e., college or university degree [58% vs. 50%]).

The families were followed with yearly person-to-person interviews, which consisted of an interview-led and a self-report part with questionnaires on a laptop. Parents usually came to local offices, but home visits were possible. They received a gift token of 200 Norwegian kroner (approximately 24 USD) for each interview. For this study, we used data from the parental interviews when the children were 12 months old. At this age, fathers were primarily invited to participate. Of 1157 parents, 61 did not respond to any of the items, and were excluded from the sample. The final sample (N = 1096) consisted of 721 fathers (65.8%) and 375 mothers (34.2%). Demographic information on children and parents is presented in Table 2.

**Table 2:** Demographic information on children and parents.

Child characteristics	
Child gender	533 girls (48.6)
Child age	12 months
Mean number of children in family	0.8 (range 0–5)
**Parent characteristics**	
Mother reports	375 (34.2)
Father reports	721 (65.8)
Mean age mother	31.4
Mean age father	33.9
Civil status mother	
Married	153 (41.2)
Cohabitants	173 (46.6)
Single /divorced/widow	45 (12.1)
Civil status father	
Married	418 (50.5)
Cohabitants	402 (48.6)
Single /divorced/widow	7 (0.8)
Main occupation mother	
Working	222 (84.7)
Social welfares/unemployed	14 (5.3)
Study	9 (3.4)
Home	17 (6.5)
Main occupation father	
Working	813 (95.6)
Social welfares/unemployed	17 (2)
Study	14 (1.6)
Home	6 (0.7)

Note. *N* = 1096. Numbers in parenthesis are percentages.

The BONDS is approved by the Regional Committee for Medical and Health Research Ethics and the Norwegian Social Sciences Data Services (approval numbers S-06067; 2009/224a). All participants provided informed written consent.

### Measurements

The Parental Stress Scale (PSS) [24] is a parent-report measure used to assess stress related to parenting. The participants’ rate the 18 items on a Likert scale ranging from 1 (“strongly disagree”) to 5 (“strongly agree”; see Table 1). The eight positive items are reversed in the coding, and a single sum score is calculated to indicate the degree of parental stress [24]. The scale was translated into Norwegian by the first author, followed by a back-translation by a bi-lingual graduate student. This back-translated version was then approved by Judy Berry, one of the authors of the PSS. Since the words, “disagree” and “agree” (used as response categories) are adverbs rather than verbs in Norwegian, a word equivalent to “some” or “a little” was added to “Disagree” and “Agree” to make a clearer distinction between the response categories “Strongly Disagree” and “Disagree”, as well as between “Agree” and “Strongly Agree”.

Parental psychological distress was assessed using a short version of the self-report symptom inventory Hopkins Symptom Checklist (SCL-8) [38], a subset of the 25-item version, which in turn is an abbreviation of the original SCL-90 [39–41]. The checklist consists of 8 items that assess psychological distress in the form of anxiety and depression (e.g., “Have little hope for the future”, “Be suddenly scared for no reason”). The respondents report their own symptom level (during the past week) on a scale ranging from one (“not at all”) to four (“extremely”). A global score is computed based on the mean. Reliability using Cronbach’s alpha was .74.

Covariates included child gender, number of siblings in the household (i.e., full sibling, half sibling, or biologically unrelated sibling), and respondent (i.e., father or mother).

### Data analyses

The total sample (N = 1096) was randomly split into two samples, using the random function in Statistical Package for the Social Sciences (SPSS, v. 35). EFA was performed on the first split-half sample (n = 543) to explore the factor structure in the data, whereas CFA was used on the second split-half sample (n = 553) to verify this structure. Both EFA and CFA were conducted using Mplus 6.1 [42], and given the ordinal nature of item responses we utilized weighted least square mean and variance (WLSMV) estimation. EFA was performed with all eighteen variables using Geomin rotation (i.e., correlated factors) to ease interpretation of factor structure, since we assumed that factors of parental stress would be correlated. To evaluate a robust and parsimonious factor structure, we used the following criteria: 1) Overall model-fit, 2) scree plot and eigenvalue > 1.0, 3) a minimum standardized factor loading of ʎ = .32 (i.e., at least 10% overlap with the factor), 4) a minimum of three indicators per factor, and 5) a simple and parsimonious factor structure. Since the chi-square (χ^2^) is sensitive to sample size, goodness-of-fit was determined by considering the root mean square error of approximation (RMSEA), the comparative fit index (CFI), the Tucker-Lewis index (TLI), and the standardized root mean square residual (SRMR). CFI and TLI range from 0 to 1, and values greater than .90/.95 are considered acceptable/excellent fit to the data [43, 44], RMSEA and SRMR values less than .06 are considered acceptable [43], but a more liberal criterion is .08 [45].

Network analysis [35, 36] was used to study connections between the different stress-items. A network consists of nodes (e.g., items) connected by edges (positive blue and negative red associations), and more strongly connected nodes are indicated by thicker edges, whereas less strongly associations have less saturated edges [46]. Given that a network often contains several arbitrarily small weights between nodes representing false positive or negative relations due to spurious connections, the adaptive LASSO penalty [47] that sets these small connections to zero, was applied. Further, the network presents partial correlations, thus connections between any of the nodes are the association left after controlling for all other connections in the network. Relying on partial correlations avoids that a correlation represented in the network is spurious, e.g., a consequence of shared variance with a third variable. More details about the estimation of networks are available elsewhere [36, 48]. Estimation of the network and centralities were conducted in JASP (version 1.1.2) [49].

## Results

### Descriptive statistics and correlations

Means, standard deviations (SD), skewness (skew), kurtosis (kurt) and correlations for the 18 PSS items are presented in Table 3. Seven items (i.e., PS01, PS05, PS06, PS07, PS14, PS17 and PS18) showed extreme values in terms of positively skewed and leptokurtic distributions (skewness ≥ 3.84 and kurtosis ≥ 13.57). All of these items, except from item PS14, were positively worded items that were reversed in line with the PSS manual [24]. The total mean was *M* = 31.00 (SD = 7.27), and thus below the scale mean. Correlations ranged from *r* = .59, p < .001 (between item PS17 and PS18) to non-significant (e.g., between item PS01 and PS03).

**Table 3:** Descriptive statistics and correlations for PSS items.

	1	2	3	4	5	6	7	8	9	10	11	12	13	14	15	16	17	18
PS01	-																	
PS02	.16**	-																
PS03	.03	-.05	-															
PS04	-.01	-.05	.42**	-														
PS05	.31**	-.15**	.02	-.02	-													
PS06	.24*	.17**	.09**	.03	.48**	-												
PS07	.19**	.10**	.08*	.03	.38**	.52**	-											
PS08	.16**	.05	.11**	.07*	.28**	.42**	.54**	-										
PS09	.07*	.02	.30**	.20**	.09**	.19**	.07*	.13**	-									
PS10	.12**	-.05	.30**	.17**	.09*	.17**	.12**	.14**	.38**	-								
PS11	.09**	.03	.20**	.16**	.10**	.09**	.08**	.13**	.28**	.32**	-							
PS12	.10**	.05	.31**	.22**	.09**	.13**	.10**	.11**	.37**	.47**	.45**	-						
PS13	.09**	.06	.19**	.14**	.11**	.15**	.09**	.14**	.29**	.21**	.19**	.25**	-					
PS14	.24**	.11**	.15**	.07**	.26**	.33**	.29**	.27**	.20**	.19**	.17**	.19**	.19**	-				
PS15	.02	.12**	.09*	.11**	.04	.05	.04	-.02	.08*	.03	.10**	.12**	.12**	.07*	-			
PS16	.14**	.10**	.23**	.12**	.16**	.23**	.17**	.19**	.33**	.37**	.33**	.39**	.39**	.35**	.17**	-		
PS17	.20**	.09*	.08**	.01	.28**	.44**	.34**	.29**	.13**	.16**	.07*	.14**	.14**	.38**	.05	.23**	-	
PS18	.20**	.13**	.01	-.01	.33**	.43**	.32**	.23**	.05*	.07*	.01	.02	.11**	.29**	-.01	.13**	.59**	-
Mean	1.24	1.39	3.11	3.01	1.12	1.12	1.17	1.34	1.94	2.77	1.75	2.09	1.32	1.13	2.69	1.66	1.10	1.05
SD	.86	1.12	1.28	1.30	.54	.45	.50	.68	1.09	1.22	1.02	1.13	.77	.53	1.52	.97	.46	.34
Skew	3.84	2.77	-.39	-.30	5.95	5.40	3.67	2.32	.87	-.12	1.10	.60	2.71	4.71	.25	1.32	6.23	9.70
Kurt	13.57	5.95	-1.11	-1.21	37.83	37.28	16.79	6.15	-.38	-1.21	.04	-.89	7.21	23.23	-1.39	.77	45.10	103.32

Note. *N* = 1096

* *p* < .05

**p >.001. Item names correspond to the ones described in Table 1. Item 1, 2, 5–8, 17 and 18 were reversed, so a higher value indicates more stress.

### Reliability

The reliability of the PSS was examined by considering the unstandardized Cronbach’s alpha (α) and McDonald’s omega (ω), of which the latter represents a less conservative and model-based estimate that does not require tau-equivalence [50–52]. For the total scale, results showed satisfactory levels of Cronbach’s alpha and McDonald’s omega (α = .74, ɷ = .79). Reliability analyses showed that item PS02 (“There is little or nothing I wouldn’t do for my child[ren] if it was necessary”) and item PS15 (“I feel overwhelmed by the responsibility of being a parent”) showed low item-total correlations (r < .20). The total scale without item PS02 (which have been omitted in most previous studies, see Table 1) resulted in an increase in reliability estimates (α = .75, ɷ = .80). When considering the original four factors [24, 29], reliabilities in the present sample were α = .70 (ɷ = .76) for Parental rewards, α = .75 (ɷ = .76) for Parental stressors, α = .33 (ɷ = .54) for Lack of control, and α = .44 (ɷ = .66) for Parental satisfaction. Thus, only Parental rewards and Parental stressors reached the acceptable benchmark value for alpha (i.e., α ≥ .70).

### Factor analyses

EFA was conducted on the first split-half sample (n = 543) using all 18 PSS items (Geomin rotation on 1–4 factors). The data did not fit a unidimensional model of parental stress (χ^2^ (135)  =  1087. 08, *p* < .001; RMSEA  =  .114 [90% CI: .108; .120], CFI = .586/TLI = .531, SRMR = .113). Inspections of the items did however reveal several low factor loadings across the multidimensional solutions (see Table 4). Especially, item PS02 and PS15 displayed loadings well below ʎ < .32, implying that these showed little influence on the parental stress construct. Consequently, the items were removed before we re-ran EFA (*N* = 16 items). In this step, eigenvalues supported a three-factor solution, but one factor appeared with only two items, and in addition, there were numerous significant cross-loadings. The two-factor solution, however, showed acceptable model-fit (χ2 (89)  =  258.70, p < .001; RMSEA  =  .059 [90% CI .051; .068], CFI = .92/TLI = .90, SRMR = .039) and provided few cross-loadings. Thus, in line with our criteria this two-factor model presented the cleanest and most parsimonious solution (see Table 4) and was chosen for further investigations.

**Table 4:** The two factor solution of the EFA with Geomin-rotated factor loadings.

# Item	Step 1		Step 2	
Factor	1	2	1	2
PS01	0.38*	0.09	0.37*	0.09
PS02	*0*.23*	*-0*.*02*	-	-
PS03	-0.01	0.49*	-0.00	0.48*
PS04	-0.09	0.41*	-0.08	0.40*
PS05	0.53*	-0.02	0.52*	-0.02
PS06	0.83*	0.04	0.83*	0.04
PS07	0.67*	-0.05	0.67*	-0.05
PS08	0.49*	0.04	0.50*	0.04
PS09	0.06	0.58*	0.06	0.59*
PS10	0.00	0.63*	0.00	0.63*
PS11	-0.02	0.57*	-0.02	0.57*
PS12	-0.00	0.75*	-0.01	0.75*
PS13	0.12*	0.34*	0.12*	0.34*
PS14	0.44*	0.21*	0.44*	0.20*
PS15	*0*.*03*	*0*.21*	-	-
PS16	0.19*	0.53*	0.19*	0.52*
PS17	0.68*	0.00	0.68*	0.00
PS18	0.75*	-0.11*	0.75*	0.10*

**Fig 1:**
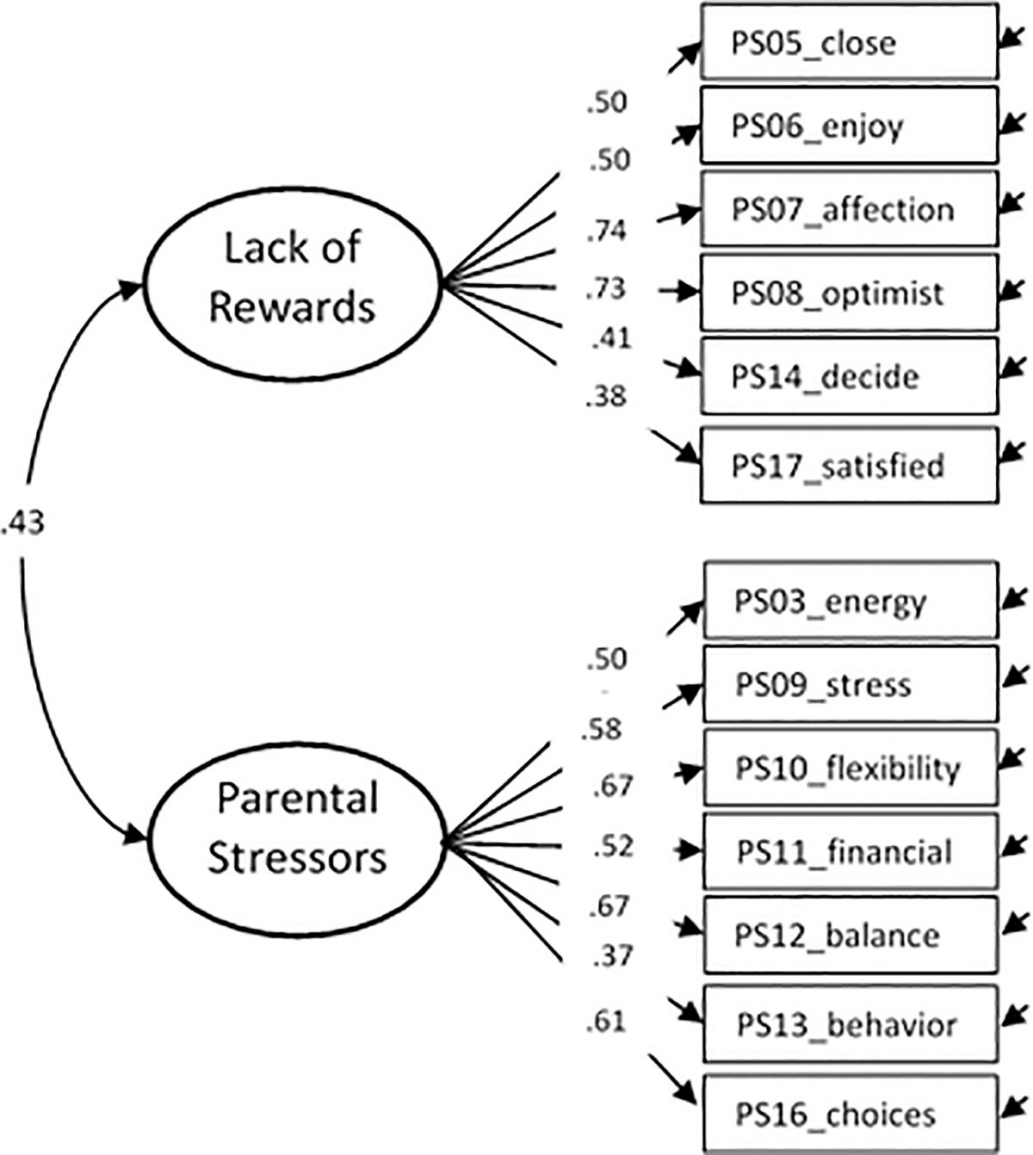
Standardized factor loadings (all p > .001) for the two-factor solution of PSS13.

CFA was applied to verify the two-factor solution, using the second split-half sample (*N* = 553). Results showed adequate model fit (χ^2^ (100)  =  271.12, *p* < .001; RMSEA  = .056 [90% CI .048; .064], CFI = .92/TLI = .91, SRMR = .051), when three residual covariances were included. Based on high modification indices (MI) between items sharing substantial overlap in content and item wording (i.e., item PS17 and PS18, PS05 and PS06, and PS03 and PS04, respectively; MI ≥ 55.13), these covariances were allowed [53]. The results did however reveal three weak loadings (ʎ ≥ .29), for item PS01, PS04, and PS18, respectively. When these items were removed (*N* = 13 PSS items) the two-factor model showed acceptable model-fit (χ^2^ (62)  =  159. 27, *p*  =  .00; RMSEA  = .053 [90% CI .043; .064], CFI = .94/TLI = .93, SRMR = .054), with item loadings ʎ ≥ .37, accounting for 52.7% of the variance (see Fig 1). The first factor reflected Lack of rewards (item PS05-PS08, PS14 and PS17) given that the majority of these items were reversed (i.e., would correspond to Parental rewards if items were not reversed). The second factor reflected Parental stressors and covered item PS03, PS09-PS13, and PS16. Reliabilities were α = .77 for Lack of rewards and α = .76 for Parental stressors (total α = .79).

### Convergent validity

Convergent validity was considered by examining the correlations between the PSS13 and parent-reported symptoms of psychological distress (anxiety and depression). Results showed significant correlations in the expected directions of small size (*r* = .19, p > .001) [54]. As shown in Table 5, the correlation was considerable lower for the Lack of rewards subscale (*r* = .08, p > .001) than for the Parental stressors (*r* = .19, p > .001).

**Table 5:** Correlations between PSS13 (total scale and subscales) and parental psychological distress (anxiety and depression).

	1		2	3	4
1. Parental stressors	-				
2. Lack of rewards	.31**	-			
3. PSS13	.94**	.62**		-	
4. Psychological distress	.19**	.08**		.19**	-

** Correlation is significant at the 0.01 level (2-tailed).

### Network analysis

**Fig 2:**
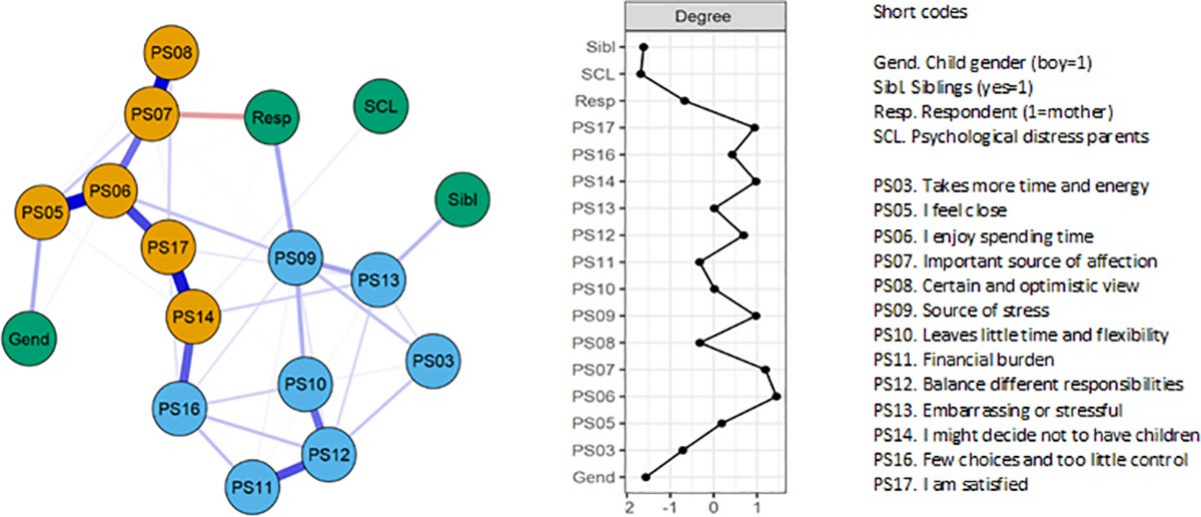
Network analysis of PSS13 and covariates. Left: Network analysis of PSS13 and covariates. Note. Blue lines represent positive connections, whereas red lines represent negative ones. Edge brightness and thickness reflect the strength of an association. Blue nodes are Parental stressors items, orange nodes are Lack of rewards items, green nodes are covariates. Right: Node degree centrality estimates.

Network analyses were applied for exploratory purposes, to reveal how the 13 different PSS items connect and interact with each other, and with the four covariates (child gender, siblings, father vs mother respondent, and psychological distress of parents). Fig 2 (left) shows the network of the 13 PSS items (in blue and orange), in addition to covariates (in green). Note that the figure depicts associations between any of the nodes after controlling for all other connections in the network. As shown, the nodes were overall positively connected. Items representing Parental stressors vs Lack of rewards clustered together, adding support to these as separate factors. Several strong connections emerged within the latter cluster, for instance between PS08 (“Having children gives me a more certain and optimistic view for the future”) and PS07 (“My child[ren] is [are] an important source of affection for me”, partial r = .44, both reversed). In addition, strong connections occurred between PS05 (“I feel close to my child[ren]”), PS06 (“I enjoy spending time with my child[ren]”), PS17 (“I am satisfied as a parent”), and PS14 (“If I had to do it over again, I might decide not to have children”, partial r = .32 - .43, all reversed except from item 14), which suggest that these items are linked together and tend to co-occur. Within the Parental stressors factor, three especially strong connections occurred between PS11 (“Having children has been a financial burden”), PS12 (“It is difficult to balance different responsibilities because of my child[ren]”), and PS10 (“Having children leaves little time and flexibility in my life”, partial r = .27 - .31), indicating that these strains tend to co-exist. Interestingly, an association between PS14 (“If I had it to do over again, I might decide not to have children”), and PS16 (“Having children has meant having too few choices and too little control over my life”), seems to bridge the two factors Lack of rewards and Parental stressors.

Notably, parents’ psychological distress (denoted SCL) appeared as a peripheral node, and did not associate with any specific node in the network. A negative association appeared between respondent’s gender and item PS07 (“My child[ren] is [are] an important source of affection for me”- reversed), in addition, having more than one child was associated with higher levels on the item “The behavior of my child(ren) is often embarrassing or stressful to me” (PS13). Finally, having a boy was positively connected with PS05 (“I feel close to my child[ren]- reversed).

The degree strength centrality (Fig 2, right) reflects a node’s activity level, i.e., the number of edges connected to the node [55]. Thus, the higher the score the more likely the node is to receive and affect other nodes in the network. The nodes with the highest degree centrality were PS07 (“My child[ren] is [are] an important source of affection for me”- reversed) and PS06 (“I enjoy spending time with my child[ren]”- reversed).

## Discussion

The purpose of this study was to investigate the validity and reliability of the Norwegian version of the PSS assessed in a large community based sample of parents with a one-year-old. We both aimed to test the psychometric properties of the Norwegian version of the PSS for the first time, and more generally add to the existing knowledge and debate about the dimensionality and properties of the scale. The results show that parental stress was best conceptualized as two separate but correlated subscales, covering altogether 13 PSS items. The factors, Parental stressors and Lack of rewards, correspond in large with previous findings, and showed internal consistencies at a satisfactory level. Convergent validity for the PSS13 was supported in that the scale correlated significantly in the expected direction with parental psychological distress (anxiety and depression). Network analyses revealed differential connections between items and covariates.

### Dimensionality of the PSS

Our findings support that the PSS taps into both stressful and satisfying experiences with being a parent, which is in line with previous research [27, 28, 32, 34]. Indeed, the combination of lack of rewards (or rewards) and demands that characterize the parental role was a major starting point for Berry and Jones [24] when creating the scale. This is probably also a reason why the PSS is a much-used alternative to other more invasive scales. As noted, the PSS is often used as a unidimensional measure, in accordance with the authors’ advice [24, 25], but without much empirical support. On the contrary, our results add to most previous research [27, 28, 32, 34] in showing that the PSS encompasses multiple dimensions of parental stress. The findings by Leung and Tsang [33] is yet the only suggesting that the scale taps into a unidimensional construct. Our findings indicate that a 13-item version of the PSS (wherein 7 items load on the Stressor dimension, and 6 on the Lack of rewards dimension) provides best fit to the present data. These results resemble those of Pontoppidan et al. [27] from Denmark, Cheung [34] from China, and Oronoz et al. [28] from Spain, which also showed that the PSS is two-dimensional, and thus that each dimension needs to be scored and interpreted separately. However, the four studies diverge with regard to both analytic strategy, number of included PSS items, and which items are removed from the scale. Still, it is interesting to note that there is fairly good consensus in what items that load on the Stressor/Strain dimension, for which altogether 5 items (i.e., PS03, PS09, PS10, PS12, and PS13) are common across all studies.

We have not been able to identify any studies that included all 18 PSS items, and obtained a robust and parsimonious factor structure. This even includes the initial validation study by Berry and Jones [24], wherein two items (PS02 and PS04) were omitted due to lack of significant factor loadings. Following our specified criteria for a robust and parsimonious factor structure, the cleanest solution consisted of 13 PSS items (i.e., deleting item PS01, PS02, PS04, PS15, and PS18). As depicted in Table 1, previous work have included from 12 [28] to 17 PSS items [34] with some variation as to which items are deleted. Importantly though, item 2 (“There is little or nothing I wouldn’t do for my child[ren] if it was necessary”) has, without exception, been omitted from the scale. This probably relates to the fact that this particular statement contains a double negation, which hampers interpretation. In addition, as pointed out by Cheung [34], the meaning is conceptually ambiguous in that it both implies a commitment to the parental role and signifies a high level of parental burden. Based on all cross-cultural empirical evidence, including the present findings, we support arguments that this item should be excluded [34]. Otherwise, decisions regarding which PSS items to keep should be based on rigorous psychometric evaluations in the relevant cultural context, using adequate sample sizes.

As regards the methodological approaches applied to testing the factor structure of the PSS, most studies have used EFA [24, 28, 34], but CFA [32] and Rasch modeling [27, 33] have also been utilized. Of the two factor analytic approaches, EFA is primarily data-driven and is most appropriate when links between the observed variables and their underlying factors are unknown, whereas CFA is theoretically grounded, and used to validate a known underlying latent structure [44]. According to Byrne [44], CFA is by far the more rigorous procedure. For example, prior research exploring the PSS [24, 28] illustrate how EFA allows all items to load freely without constraints. That is, the same items are permitted to load on several factors, without a minimum loading or number of items per factor. In the four-factor solution reported by Berry and Jones [24], several cross-loadings appear. In particular, item PS16 is included in the factors Parental stressors and Loss of control and item PS18 is included in the factors Parental lack of rewards and Parental satisfaction. As noted [27, 34], this factorial solution also involves conceptual overlap between some of the factors and superfluous subdivisions of the main dimensions (i.e., Parental lack of rewards and Parental stressors). In line with this, the lacking mechanism for identifying which areas of an EFA model that contributes to the misfit of the model, is clearly unfortunate [44].

To determine the optimal number of factors across PSS items, we combined EFA and CFA methodology. This approach was considered better than merely using CFA to test all previously suggested solutions (see Table 1), given that: (a) the solutions include different number of items, (b) items were excluded for different reasons (e.g., non-significant or small factor loadings, extensive skewness, wording, cultural reasons), (c) the criteria used for inclusion/exclusion of items varies across studies, and (d) these criteria are poorly documented. Still, we cannot rule out the possibility that solutions other than our two-factor model could represent the data well, if alternative criteria were used. For example, one of our criteria was that a factor should have a minimum of three indicators, since those with only two indicators are more prone to estimation problems, and generally considered weaker and more unstable [56, 57]. In addition, for reliability, shortness of items in a scale is of major concern [58].

### Reliability and convergent validity of the PSS

It is noteworthy that the decrease in reliability for the total PSS13 was only minimal as compared to the complete PSS18 version. Thus, the shorter scale is as good as the original. In addition, our final two-dimensional solution provided factors with acceptable reliabilities. When testing the internal consistency of the four original factors [24] in our data, the result mirrored those of Berry and Jones in showing particularly poor reliabilities for two of the factors (i.e., Loss of control and Parental satisfaction), each having three indicators. It should also be noted that these alphas are biased since two items appeared in the same factors. The implications of using factors with low reliabilities are crucial, resulting in for instance poor validity, and attenuated interpretations and effect sizes [58]. Overall, these results clearly indicate that the two factors Loss of control and Parental satisfaction as presented by Berry and Jones [24] need to be improved, for instance through considering other items or increase the number of items.

Our results show that the PSS was significantly associated with parental psychological distress, in the expected direction. The relation was of small size, but this was expected given that this is a population based and non-clinical sample. In comparison, the results from Oronoz et al. [28] indicated higher correlations and thus a stronger relation between their revised PSS12 scale and parental symptoms of anxiety and depression among first-time parents with a child aged 3–8 months.

### The network of PSS

We applied network analysis to explore the connections between the PSS items and four relevant covariates. Compared to a latent approach, where items are supposed to be reflective and interchangeable indicators on a latent construct, the network methodology consider parental stress to constitute the associations between items. As such, the network of PSS items provides an additional perspective on parental stress to the latent approach The items were overall positively connected, and items within Parental stressors and Lack of rewards clustered, which add support to these as separate factors. Differential strengths between the PSS nodes were apparent. Within the cluster Lack of rewards (reversed positive items), strong connections appeared between the majority of items, showing that these tend to co-occur. In the cluster Parental stressors, the strongest edges were found between item PS10 (“Little time and flexibility”), PS11 (“Financial burden”) and PS12 (“Balance different responsibilities”). Thus, these three stressors, which all reflect common challenges with having children, tend to appear together. Interestingly, parents’ symptoms of mental distress did not connect strongly to any particular item, indicating that the association probably reflects a relation between the overall burden of parental stress and mental distress. The only negative connection apparent in the network was between Resp (respondent, 1 = mother/0 = father) and item PS07 (“important source of affection”- reversed), implying that mothers evaluated lower levels on this reversed item. Thus, mothers seem to perceive their children as a greater source of affection than did fathers. Moreover, child gender (boy = 1/girl = 0) related to item PS05 (“I feel close”- reversed), meaning that parents report feeling less close to boys compared to girls. However, this result may be due to the unequal number of mothers and fathers in our sample. The degree estimate was investigated to get information about the importance of the different stress items [59]. The nodes with the highest strength index were PS06 (“I enjoy spending time with my child[ren]”-reversed), PS07 (“My child[ren] is [are] an important source of affection for me”—reversed), PS17 (“I am satisfied as a parent”—reversed), and PS09 (“The major source of stress in my life is my child[ren]”). Thus, the results indicate that these items are the most important in the network constituting parental stress, i.e., they have strong connections to many of the other nodes. Consequently, they represent important target items when it comes to identifying and helping parents who struggle with stress in the parent role.

### Strengths and limitations

Several strengths and limitations pertain to this study. First, the study contributes with a rigorous psychometric test of the Norwegian version of the PSS using a large, population based sample from a demographically diverse population. The combination of the sample size, pre-defined criteria for item selection, and the complementary use of EFA and CFA holds clear advantages as compared with some prior research. Still, the sample is somewhat biased towards families with better sociodemographic backgrounds when compared with the Norwegian population. Further, we did not investigate parental stress among mothers and fathers, separately. It may be that perceived parental stress differs between mothers and fathers and this should be interesting to address in future studies. Finally, we excluded items that showed non-significant or low factor loadings (i.e., λ < .32). Noteworthy, other studies have deleted items based on different criteria, e.g., extensive skewness or challenges related to translations. Consequently, more research is advocated to establish consensus about the psychometric properties of the PSS, to make it a valid and reliable instrument for use in research and practice.

## Conclusions and practical implications

Given that our sample was recruited from the normal population, it is not surprising that the level of parental stress was not particularly high. Unfortunately, there is no established cut-off values for the PSS. This was pointed out by Louie and colleagues [25] and has also been highlighted by others [27, 29]. Future studies should strive to establish appropriate clinical cut-off scores, indicative of clinically relevant levels of parenting stress, preferably by conducting sensitivity-specificity studies. However, given the numerous versions of the PSS this is a challenging task. Lastly, it should be noted that the different practices across studies to reverse the positively worded items (i.e., in accord with Berry and Jones [24]) or not, result in a somewhat confusing and inconsistent practice to denote the corresponding dimension either Lack of rewards (when reversing the items) or Rewards (when not reversing).

Summing up, the present study adds to the existing pool of literature on the psychometric properties of the PSS. In practice, our findings are both encouraging and cautionary. First, the results support that the scale taps into both stressful and unsatisfying experiences with being a parent, and that the measure is multidimensional in nature (Parental stressors and Lack of rewards). Second, in line with previous work, no adequate fit could be established for the original 18 items version of the scale, even less when considering the reliabilities of the factors originally presented. Our results revealed several items with non-significant or low factor loadings. In particular, item PS02 consistently produces non-significant loadings, and is also hard to understand. Thus, item PS02 should be excluded from the scale. Third, the psychometric properties of the Norwegian 13-item version of the PSS in the form of reliability, construct validity, and convergent validity are satisfactory.

A valid and reliable measurement is essential to identify parental stress in the population. Our results suggest that practitioners should be aware that PSS is a two-dimensional instrument, and that several of the items have shown to be poor indicators of stress. Moreover, the finding that some of the items seem to be more central than others in the network constituting parental stress and therefore represent important target items when it comes to identifying and helping parents who struggle with stress relating to child rearing and caregiving, is also informative for practioners.
